# Prudent Antimicrobial Use Is Essential to Prevent the Emergence of Antimicrobial Resistance in *Yersinia enterocolitica* 4/O:3 Strains in Pigs

**DOI:** 10.3389/fmicb.2022.841841

**Published:** 2022-03-10

**Authors:** Juho Koskinen, Pilar Ortiz-Martínez, Riikka Keto-Timonen, Suvi Joutsen, Maria Fredriksson-Ahomaa, Hannu Korkeala

**Affiliations:** Department of Food Hygiene and Environmental Health, Faculty of Veterinary Medicine, University of Helsinki, Helsinki, Finland

**Keywords:** antibiotic, antimicrobial, antimicrobial policy, control, foodborne pathogen, pig, resistance, *Yersinia enterocolitica*

## Abstract

*Yersinia enterocolitica* is a psychrotrophic zoonotic foodborne pathogen. Pigs are considered the main reservoir of *Y. enterocolitica* 4/O:3, which is the most commonly isolated bioserotype in many European countries. Consuming pork contaminated with *Y. enterocolitica* can be a health threat, and antimicrobial-resistant strains may complicate the treatment of the most severe forms of yersiniosis. We analyzed the antimicrobial resistance of 1,016 pathogenic porcine *Y. enterocolitica* 4/O:3 strains originating from Belgium, Estonia, Finland, Germany, Italy, Latvia, Russia, Spain, and the United Kingdom. Based on available reports, we also compared antimicrobial sales for food production animals in these countries, excluding Russia. Antimicrobial resistance profiles were determined using a broth microdilution method with VetMIC plates for 13 antimicrobial agents: ampicillin, cefotaxime, ceftiofur (CTF), chloramphenicol (CHL), ciprofloxacin, florfenicol, gentamicin, kanamycin, nalidixic acid (NAL), streptomycin (STR), sulfamethoxazole (SME), tetracycline (TET), and trimethoprim (TMP). The antimicrobial resistance of *Y. enterocolitica* 4/O:3 strains varied widely between the countries. Strains resistant to antimicrobial agents other than ampicillin were rare in Estonia, Finland, Latvia, and Russia, with prevalence of 0.7, 0.4, 0, and 8.3%, respectively. The highest prevalence of antimicrobial resistance was found in Spanish and Italian strains, with 98 and 61% of the strains being resistant to at least two antimicrobial agents, respectively. Resistance to at least four antimicrobial agents was found in 34% of Spanish, 19% of Italian, and 7.1% of English strains. Antimicrobial resistance was more common in countries where the total sales of antimicrobials for food production animals are high and orally administered medications are common. Our results indicate that antimicrobials should be used responsibly to treat infections, and parenteral medications should be preferred to orally administered mass medications.

## Introduction

*Yersinia enterocolitica* is a foodborne pathogen capable of causing yersiniosis, the fourth most reported bacterial zoonosis in the European Union [European Food Safety Authority and European Centre for Disease Prevention and Control (EFSA and ECDC), 2021]. Pigs are the main reservoir of pathogenic *Y. enterocolitica*, especially bioserotype 4/O:3, and these bacteria have frequently been isolated from the tonsils and feces of clinically healthy pigs (Gürtler et al., 2005; Fredriksson-Ahomaa et al., 2007; Laukkanen et al., 2009; Ortiz Martínez et al., 2009, 2011; Virtanen et al., 2014; Koskinen et al., 2019). Consequently, pork products are important sources of human yersiniosis (Tauxe et al., 1987; Ostroff et al., 1994; Fredriksson-Ahomaa et al., 2006, 2010a; Virtanen et al., 2013).

Yersiniosis usually manifests as gastroenteritis, but the infection may, for example, cause pseudoappendicitis or sepsis or lead to immunological sequelae such as reactive arthritis or erythema nodosum (Bottone, 1999; Fredriksson-Ahomaa et al., 2010a). Most infections are self-limiting, and antimicrobial therapy is therefore not needed. Antimicrobial agents, such as fluoroquinolones, trimethoprim-sulfamethoxazole (SME), tetracycline (TET), and third-generation cephalosporins, are warranted for more severe infections, in which mortality can rise to 50%, and for severe postinfectious complications (Crowe et al., 1996; Jiménez-Valera et al., 1998; Hoogkamp-Korstanje et al., 2000; Guinet et al., 2011; Fàbrega and Vila, 2012). *Y. enterocolitica* has intrinsic resistance to many β-lactam antibiotics, such as penicillin, ampicillin, and first-generation cephalosporins, due to the presence of two β-lactamase genes *blaA* and *blaB* (Cornelis and Abraham, 1975; Bent and Young, 2010; Bonke et al., 2011).

Antimicrobial resistance is a concerning global health threat [World Health Organization (WHO), 2015]. If necessary actions are not taken, an estimated 10 million people could die in 2050 due to antimicrobial resistance with massive consequences for patients, healthcare systems, and economies (Dadgostar, 2019). Using antimicrobial agents for livestock is a significant part of this multifactorial problem and may increase the antimicrobial resistance of foodborne pathogens. These pathogens may transmit from production animals to humans via food products, water, or by direct contact (Collignon, 2012). The transmission of antimicrobial-resistant porcine *Y. enterocolitica* strains to humans may complicate the treatment of the most severe forms of yersiniosis or other bacterial infections. To control this public health threat, regular and comprehensive monitoring of antimicrobial resistance is required worldwide, so that necessary actions can be taken.

The aim of our study was to assess the antimicrobial resistance of 1,016 *Y. enterocolitica* bioserotype 4/O:3 strains isolated from porcine origin in nine European countries, and to compare the use of antimicrobial agents in these countries. We observed wide variation in antimicrobial resistance. Concerning levels of antimicrobial resistance were found in countries where the total use of antimicrobial agents is high, especially Spain and Italy. Our results indicate that prudent use of antimicrobials is essential to control antimicrobial resistance already at the farm level.

## Materials and Methods

### Strains

A total of 1,016 strains of *Y. enterocolitica* pathogenic bioserotype 4/O:3 from nine European countries (Belgium, Estonia, Finland, Germany, Italy, Latvia, Russia, Spain, and the United Kingdom) were studied. *Yersinia enterocolitica* strains originated from pork and were chosen from the culture collection of the Department of Food Hygiene and Environmental Health (University of Helsinki, Helsinki, Finland). The strains had been isolated between 1999 and 2007 (Supplementary Table 1) from pig tonsils, except for eight of the German strains that were isolated from the tongue (*n* = 4), surface samples of pig carcasses (*n* = 3), and head meat (*n* = 1).

### Antimicrobial Resistance Testing

Antimicrobial resistance was tested using a broth microdilution method according to the standards of the Clinical and Laboratory Standards Institute (CLSI) (2017). The following 13 antimicrobials were tested: ampicillin (concentration range 0.25–32 mg/L), cefotaxime (0.06–2 mg/L), ceftiofur (CTF; 0.12–16 mg/L), chloramphenicol (CHL; 1–128 mg/L), ciprofloxacin (0.008–1 mg/L), florfenicol (4–32 mg/L), gentamicin (0.5–64 mg/L), kanamycin (2–16 mg/L), nalidixic acid (NAL; 1–128 mg/L), streptomycin (STR; 2–256 mg/L), sulfamethoxazole (16–2,048 mg/L), tetracycline (0.5–64 mg/L), and trimethoprim (TMP; 0.25–32 mg/L). Susceptibility monitoring was performed on VetMIC plates (SVA, Uppsala, Sweden). The strains were grown on *Yersinia* selective agar base (Oxoid Ltd., Basingstoke, New Hampshire, United Kingdom) at 30°C for 24 h. Four or five colonies were transferred into 5 ml of Müller-Hinton II broth (BBL, Müller-Hinton II broth, cation adjusted; Beckton, Dickinson and Company, Sparks, MD, United States) and incubated at 30°C until the absorbance of the broth was 0.08–0.1, to obtain an inoculum size of 10^8^ CFU/ml. The inoculums for broth microdilution were prepared by mixing 10 μl of the inoculum to 10 ml of Müller-Hinton II broth. Each well of the VetMIC plate was filled with 50 μl of the inoculum and sealed with covering tape. The plates were incubated for 1 h in a shaker (150 rpm) and 16–18 h at 30°C. *Escherichia coli* ATCC 25922 was used as a control strain and incubated at 37°C.

The plates were evaluated with visual examination using a magnifying mirror. The minimum inhibitory concentration (MIC) was defined as the lowest antimicrobial concentration that inhibited bacterial growth. The strains were categorized as susceptible (S), intermediately resistant (I), or resistant (R). Clinical breakpoints for *Enterobacteriaceae* from the Clinical and Laboratory Standards Institute (CLSI) (2017) were used, except for ceftiofur, florfenicol, and streptomycin, for which we used the breakpoints for *Salmonella* spp. of Danish Integrated Antimicrobial Resistance Monitoring and Research Program (DANMAP) (2015). The results were analyzed using WHONET 5.6 software (Stelling and O'Brien, 1997). In our study, a strain was classified as multiresistant if it was resistant to at least two of the antimicrobial agents tested, excluding ampicillin, which shows frequent intrinsic resistance.

### Estimations of Antimicrobial Use in European Countries

We evaluated the antimicrobial use and policies of eight countries (Belgium, Estonia, Finland, Germany, Italy, Latvia, Spain, and the United Kingdom) based on the European Surveillance of Veterinary Antimicrobial Consumption (ESVAC) sales report by the European Medicines Agency (EMA) for year 2011 [European Medicines Agency (EMA), 2013], as no direct data are available for the actual use of antimicrobial agents. This report was the first to cover all eight countries. Respective data were not available from Russia.

In the ESVAC reports, sales are measured in relation to the estimated quantity of animal biomass, i.e., the antimicrobials used for food production animals in milligrams per population correction unit (mg/PCU). The PCU is a technical unit of measurement, which is used to estimate the mass of treated livestock and slaughtered animals during a year, and animals exported or imported for slaughter or fattening in another member country are also taken into account [European Medicines Agency (EMA), 2013]. In addition, we compared the use of orally administered and injectable veterinary antimicrobials based on the corresponding proportions of the sales in each country. Data from the first Joint Interagency Antimicrobial Consumption and Resistance Analysis (JIACRA) report by the European Centre for Disease Prevention and Control, European Food Safety Authority, and European Medicines Agency (ECDC, EFSA, and EMA) (2015) were also used to measure antimicrobial use for food production animals and as a baseline for humans.

A report by the European Agency for the Evaluation of Medicinal Products (EMEA) (1999) was used to estimate the use of all antimicrobials, therapeutic antimicrobials, and growth promoter antimicrobials for animals in the mid-1990s. Antimicrobial use in Belgium and Luxemburg, Finland, Germany, Italy, Spain, and the United Kingdom in 1997 was made proportional to the number of animals slaughtered (mg/kg) in these countries in 1996. These data represent antimicrobial use in the countries in the mid-1990s, a decade before antimicrobial growth promoters were banned in the European Union (EU) in 2006 [European Union (EU), 2005]. The data regarding Belgium include Luxemburg, and in Finland, growth promoter antimicrobial levels were less than 2 mg/kg, but 2 mg/kg was used for the analyses.

### Statistical Analyses

Statistical analyses were performed in IBM SPSS Statistics 27 (IBM, Armonk, NY, United States). One-tailed Pearson’s correlation was used to measure correlations between the observed prevalence of antimicrobial resistance and the different estimations of antimicrobial use, that is, total, oral, and injectable antimicrobial sales in mg/PCU, growth promoter sales, as well as proportions of antimicrobial sales by administration route. In addition, countries were categorized into two groups based on whether more than two thirds of antimicrobial sales were oral medications instead of parenteral medications such as injections, and antimicrobial resistance between the two groups was compared with the Student’s *t* test with unequal variances.

## Results

### Antimicrobial Resistance of *Yersinia enterocolitica*

All strains were susceptible to cefotaxime, ceftiofur, ciprofloxacin, florfenicol, gentamicin, and kanamycin (Table 1). The MICs are shown in Supplementary Table 2. Estonian, Finnish, and Latvian strains were susceptible to all other antimicrobials than ampicillin, excluding one Estonian strain resistant to trimethoprim and one Finnish strain resistant to sulfamethoxazole. Resistance to streptomycin was found frequently, with highest resistance levels in Spain (98%), Italy (62%), and Belgium (55%). Resistance to sulfamethoxazole was also common, with highest prevalence of resistance in Spanish (99%), English (82%), and Italian (61%) strains. Tetracycline resistance was found in 49% of Italian and 27% of Spanish strains. Trimethoprim resistance was most common among Italian (30%) and English (21%) strains. Resistance to chloramphenicol and nalidixic acid was found in Spanish strains only.

**Table 1:** Number of porcine *Yersinia enterocolitica* 4/O:3 strains resistant to antimicrobials. All strains were susceptible to cefotaxime^a^, ceftiofur^b^, ciprofloxacin^c^, florfenicol^d^, gentamicin^e^, and kanamycin^f^.

Country (number of strains)	Ampicillin^g^				Chloramphenicol^h^				Nalidixic acid^i^			Streptomycin^j^			Sulfamethoxazole^k^			Tetracycline^l^				Trimethoprim^m^		
	S	I	R	% (CI)^n^	S	I	R	% (CI)^n^	S	R	% (CI)^n^	S	R	% (CI)^n^	S	R	% (CI)^n^	S	I	R	% (CI)^n^	S	R	% (CI)^n^
Belgium (94)	0	9	85	90 (82–95)	94	0	0	0 (0–4.9)	94	0	0 (0–4.9)	42	52	55 (45–65)	92	2	2.1 (0.4–8.2)	94	0	4	4.3 (1.4–11)	94	0	0 (0–4.9)
Estonia (143)	0	0	143	100 (97–100)	143	0	0	0 (0–3.3)	143	0	0 (0–3.3)	143	0	0 (0–3.3)	143	0	0 (0–3.3)	143	0	0	0 (0–3.3)	142	1	0.7 (0–4.4)
Finland (233)	0	17	216	93 (88–96)	233	0	0	0 (0–2.0)	233	0	0 (0–2.0)	233	0	0 (0–2.0)	232	1	0.4 (0–2.7)	233	0	0	0 (0–2.0)	233	0	0 (0–2.0)
Germany (98)	0	0	98	100 (95–100)	98	0	0	0 (0–4.7)	98	0	0 (0–4.7)	92	6	6.1 (2.5–13)	94	4	4.1 (1.3–11)	98	0	0	0 (0–4.7)	98	0	0 (0–4.7)
Italy (105)	0	24	81	77 (68–85)	104	1	0	0 (0–4.4)	105	0	0 (0–4.4)	40	65	62 (52–71)	41	64	61 (51–70)	42	12	51	49 (39–59)	73	32	30 (22–40)
Latvia (70)	0	0	70	100 (94–100)	70	0	0	0 (0–6.5)	70	0	0 (0–6.5)	70	0	0 (0–6.5)	70	0	0 (0–6.5)	70	0	0	0 (0–6.5)	70	0	0 (0–6.5)
Russia (60)	0	5	55	92 (81–97)	60	0	0	0 (0–7.5)	60	0	0 (0–7.5)	57	3	5.0 (1.3–15)	59	1	1.7 (0.1–10)	59	0	1	1.7 (0.1–10)	60	0	0 (0–7.5)
Spain (185)	0	0	185	100 (98–100)	20	0	165	89 (84–93)	166	19	10 (6.5–16)	4	181	98 (94–99)	2	183	99 (97–100)	134	1	50	27 (21–34)	185	0	0 (0–2.5)
UK (28)	0	1	27	96 (80–100)	28	0	0	0 (0–15)	28	0	0 (0–15)	26	2	7.1 (1.2–25)	5	23	82 (62–93)	26	0	2	7.1 (1.2–25)	22	6	21 (9–41)

a MIC < 2 mg/L susceptible (S), 2 mg/L intermediately resistant (I), and >2 mg/L resistant (R); tested concentration range 0.06–2 mg/L.

b MIC < 4 mg/L susceptible (S), and ≥4 mg/L resistant (R); tested concentration range 0.12–16 mg/L.

c MIC < 2 mg/L susceptible (S), 2 mg/L intermediately resistant (I), and >2 mg/L resistant (R); the breakpoints were higher than the tested concentration range 0.008–1 mg/L.

d MIC < 32 mg/L susceptible (S), and ≥32 mg/L resistant (R); tested concentration range 4–32 mg/L.

e MIC < 8 mg/L susceptible (S), 8 mg/L intermediately resistant (I), and >8 mg/L resistant (R); tested concentration range 0.5–64 mg/L.

f MIC < 32 mg/L susceptible (S), 32 mg/L intermediately resistant (I), and >32 mg/L resistant (R); the breakpoints were higher than the tested concentration range 2–16 mg/L.

g MIC < 16 mg/L susceptible (S), 16 mg/L intermediately resistant (I) and >16 mg/L resistant (R); tested concentration range 0.25–32 mg/L.

h MIC < 16 mg/L susceptible (S), 16 mg/L intermediately resistant (I), and > 16 mg/L resistant (R); tested concentration range 1–128 mg/L.

i MIC < 32 mg/L susceptible (S), and ≥32 mg/L resistant (R); tested concentration range 1–128 mg/L.

j MIC < 32 mg/L susceptible (S), and ≥32 mg/L resistant (R); tested concentration range 2–256 mg/L.

k MIC < 512 mg/L susceptible (S), and ≥512 mg/L resistant (R); tested concentration range 16–2048 mg/L.

l MIC < 8 mg/L susceptible (S), 8 mg/L intermediately resistant (I), and >8 mg/L resistant (R); tested concentration range 0.5–64 mg/L.

m MIC < 16 mg/L susceptible (S), and ≥16 mg/L resistant (R); tested concentration range 0.25–32 mg/L.

n Percentage of resistant strains for an antimicrobial agent and CIs (95% confidence level).

### Antimicrobial Resistance to at Least Two Antimicrobial Agents

Resistance to multiple antimicrobials was found especially in Spanish, Italian, and English *Y. enterocolitica* 4/O:3 strains, and in a few Belgian strains (Table 2). The majority of the Spanish strains (98%) were multiresistant, and the most common antimicrobial resistance pattern was CHL-STR-SME (Table 3). Multiresistance patterns CHL-STR-SME-TET and CHL-NAL-STR-SME were found in 23 and 6.0% of Spanish strains, respectively. More than half of the Italian strains (61%) were multiresistant, while 21, and 5.3% of the English, and Belgian strains were resistant to at least two antimicrobials, respectively. Resistance to four or five antimicrobials was observed in Spanish (*n* = 62), Italian (*n* = 20), and English (*n* = 2) strains.

**Table 2:** Antimicrobial resistance of porcine *Yersinia enterocolitica* 4/O:3 strains in nine European countries.

Country (number of strains)	Total number of resistant^a^ strains (%)	Number of strains *n* (%) resistant^a^ to					Total number of multiresistant^b^ strains (%)
		One antimicrobial	Two antimicrobials	Three antimicrobials	Four antimicrobials	Five antimicrobials	
Belgium (94)	53 (56%)	48 (51%)	5 (5.3%)	0 (0%)	0 (0%)	0 (0%)	5 (5.3%)
Estonia (143)	1 (0.70%)	1 (0.70%)	0 (0%)	0 (0%)	0 (0%)	0 (0%)	0 (0%)
Finland (233)	1 (0.43%)	1 (0.43%)	0 (0%)	0 (0%)	0 (0%)	0 (0%)	0 (0%)
Germany (98)	10 (10%)	10 (10%)	0 (0%)	0 (0%)	0 (0%)	0 (0%)	0 (0%)
Italy (105)	65 (62%)	1 (0.9%)	1 (0.9%)	43 (41%)	20 (19%)	0 (0%)	64 (61%)
Latvia (70)	0 (0%)	0 (0%)	0 (0%)	0 (0%)	0 (0%)	0 (0%)	0 (0%)
Russia (60)	5 (8.3%)	5 (8.3%)	0 (0%)	0 (0%)	0 (0%)	0 (0%)	0 (0%)
Spain (185)	184 (99%)	3 (1.6%)	16 (8.6%)	103 (56%)	55 (30%)	7 (3.8%)	181 (98%)
United Kingdom (28)	23 (82%)	17 (61%)	4 (14%)	0 (0%)	2 (7.1%)	0 (0%)	6 (21%)
Total	342 (34%)	86 (8.5%)	26 (2.6%)	146 (14.4%)	77 (7.6%)	7 (0.7%)	256 (25%)

a Ampicillin excluded.

b Strains resistant to at least two antimicrobials, excluding ampicillin.

**Table 3:** Antimicrobial multi-resistance patterns of porcine *Yersinia enterocolitica* 4/O:3 strains.

Antimicrobial multi-resistance pattern^a^	Number of strains (%)	Countries^b^ showing pattern (*n*)
STR-SME	18 (1.8%)	BE (1), ES (16), IT (1)
STR-TET	4 (0.4%)	BE (4)
SME-TMP	4 (0.4%)	UK (4)
CHL-STR-SME	103 (10%)	ES (103)
STR-SME-TET	31 (3.1%)	IT (31)
STR-SME-TMP	12 (1.2%)	IT (12)
CHL-STR-SME-TET	43 (4.2%)	ES (43)
STR-SME-TET-TMP	22 (2.2%)	IT (20), UK (2)
CHL-NAL-STR-SME	11 (1.1%)	ES (11)
CHL-CTF-STR-SME	1 (0.1%)	ES (1)
CHL-NAL-STR-SME-TET	7 (0.7%)	ES (7)

a Ampicillin (AMP), cefotaxime (CTX), ceftiofur (CTF), chloramphenicol (CHL), ciprofloxacin (CIP), florfenicol (FLO), gentamicin (GEN), kanamycin (KAN), Nalidixic acid (NAL), streptomycin (STR), sulfamethoxazole (SME), tetracycline (TET), and trimethoprim (TMP).

b BE, Belgium; ES, Spain; IT, Italy; and UK, United Kingdom.

### Use of Antimicrobial Agents and Its Correlation With Antimicrobial Resistance

**Figure 1:**
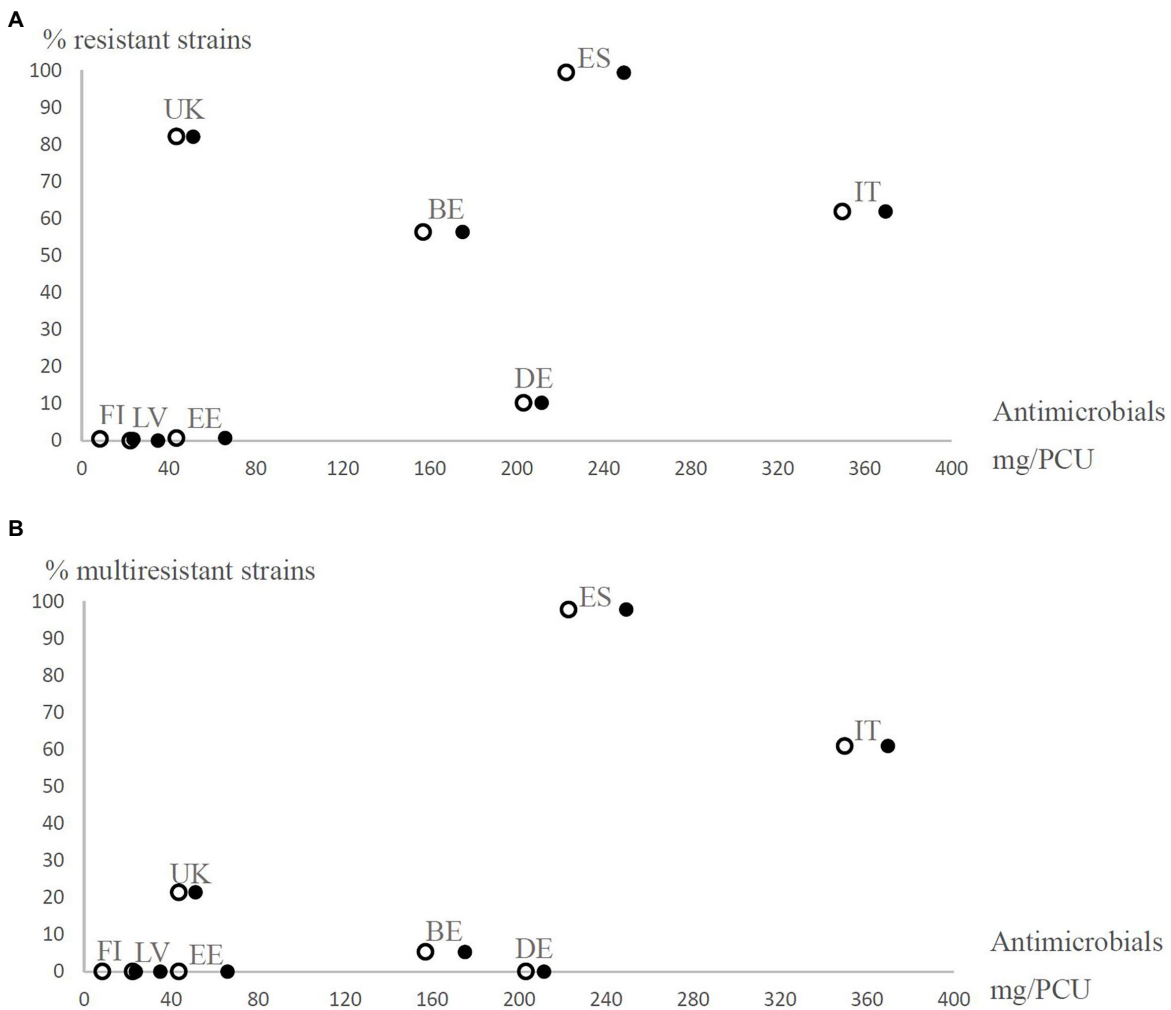
Prevalence of resistant (resistance to at least one antimicrobial agent excluding ampicillin; **A**) and multiresistant (resistance to at least two antimicrobial agents excluding ampicillin; **B**) *Yersinia enterocolitica* 4/O:3 strains from different European countries (BE, Belgium; DE, Germany; EE, Estonia; ES, Spain; FI, Finland; IT, Italy; LV, Latvia; and UK=United Kingdom) observed in the present study in relation to the estimated number of antimicrobials sold for treating production animals in mg per population correction unit (mg/PCU) in 2011. Open circles represent orally administered antimicrobials and closed circles represent all antimicrobials. The proportions of oral solutions, oral powders, and premixes as percentages of total sales of antimicrobial agents in each country [European Medicines Agency (EMA), 2013] were used as coefficients to estimate how many mg/PCU of antimicrobials were administered orally for production animals.

**Figure 2:**
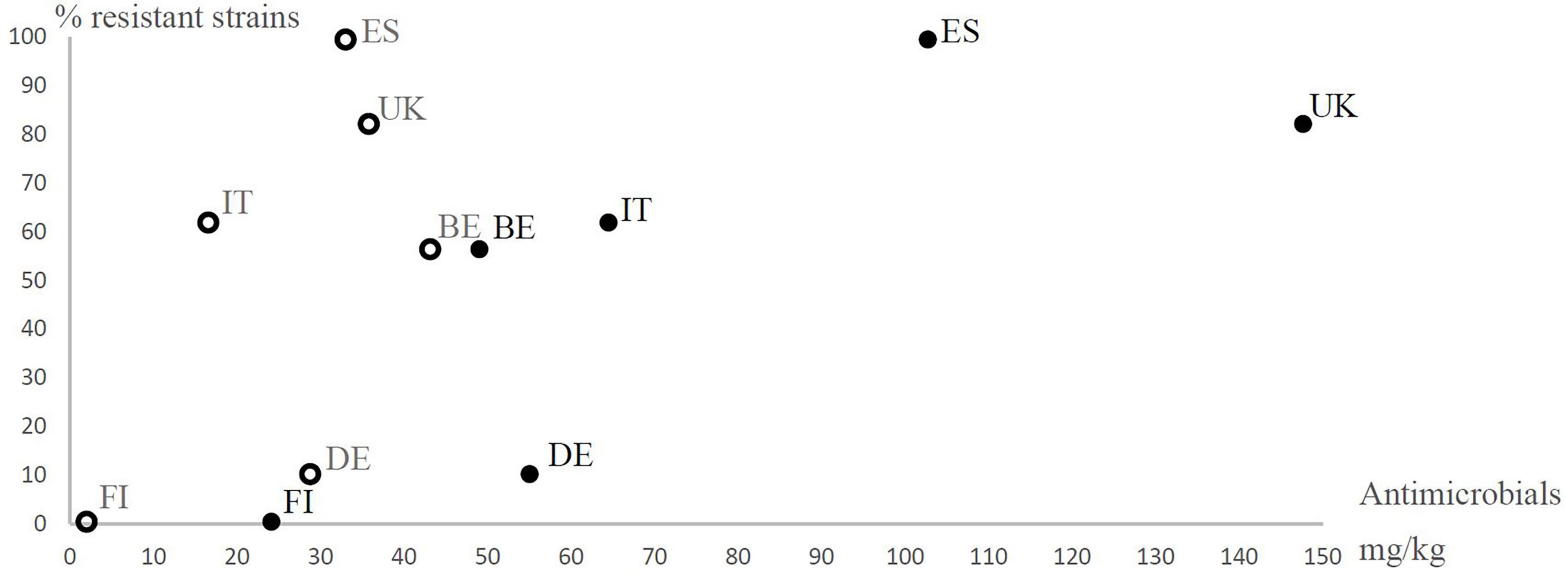
Prevalence of antimicrobial resistant (resistant to at least one antimicrobial agent excluding ampicillin) *Yersinia enterocolitica* 4/O:3 strains in different European countries (BE, Belgium; DE, Germany; ES, Spain; FI, Finland; IT, Italy; and UK, United Kingdom) observed in the present study in relation to the estimated number of growth promoter antimicrobials used (open circles) and the therapeutic antimicrobials used (closed circles) for animals (mg/kg) in six European countries in the mid-1990s. The use of antimicrobial agents for animals in mg per kg was calculated from the estimated numbers of antimicrobial agents sold for the treatment of animals in 1997 and the estimated weights of slaughtered animals in 1996 in each country. The data are from European Agency for the Evaluation of Medicinal Products (EMEA) (1999). The data regarding Belgium include Luxemburg. In Finland, growth promoter antimicrobial levels were less than 2 mg/kg, but 2 mg/kg was used for the analyses.

Based on sales data, we observed vast differences in the use of antimicrobial agents in the eight countries (Table 4). In general, higher levels of antimicrobials were used in countries, where antimicrobial resistance and multiresistance were frequent (Figures 1, 2). The prevalence of antimicrobial resistance of *Y. enterocolitica* in the countries significantly correlated with antimicrobial multiresistance (Table 5).

**Table 4:** Estimated antimicrobial use for food production animals, and for humans as a baseline, in eight European countries based on available reports.

	Country							
	Belgium	Estonia	Finland	Germany	Italy	Latvia	Spain	UK
Antimicrobial sales in 2011^a^								
Total (mg/PCU^b^)	175.2	66.0	23.8	211.5	369.7	35.0	249.4	51.2
Oral powders, oral solutions, and premixes								
Sales (mg/PCU)	157.0	43.6	8.4	203.2	349.9	22.4	222.9	43.6
Proportion of total sales	90%	66%	35%	96%	95%	64%	89%	85%
Injections								
Sales (mg/PCU)	17.3	20.0	13.9	6.7	18.7	9.9	10.8	6.3
Proportion of total sales	9.9%	30%	58%	3.2%	5.1%	28%	4.3%	12%
Proportion of pigs in PCU	55%	30%	35%	47%	22%	19%	47%	11%
Antimicrobial consumption in 2012^c^								
Consumption in hospitals included	Yes	Yes	Yes	No	Yes	Yes	No	No
Consumption (mg/kg biomass)								
Humans	162.6	70.1	140.1	66.9	167.1	88.8	108.6	104.2
Production animals	161.1	56.0	23.8	204.8	341.0	44.1	242.0	66.3
Antimicrobials sold in 1997 (mg) divided by production animals slaughtered in 1996 (kg)^d^								
Total (mg/kg)	92.2^e^	no data	24.1	83.7	81.3	no data	135.8	183.5
Therapeutic antimicrobials (mg/kg)	49.1	no data	24.1	55.1	64.5	no data	102.6	147.7
Growth promoter antimicrobials	43.2	no data	< 2	28.8	16.6	no data	33.0	35,8

a Data from ESVAC report [European Medicines Agency (EMA), 2013].

b PCU, population correction unit.

c Data from JIACRA report [European Centre for Disease Prevention and Control, European Food Safety Authority, and European Medicines Agency (ECDC, EFSA, and EMA), 2015].

d Data from European Agency for the Evaluation of Medicinal Products (EMEA) (1999).

e Data from Belgium includes Luxemburg.

**Table 5:** Correlation between antimicrobial use and resistance of porcine *Yersinia enterocolitica* 4/O:3 strains in Europe.

Antimicrobial use	Prevalence of antimicrobial resistance of *Y. enterocolitica* 4/O:3							
	Resistance^a^ to at least one antimicrobial				Resistance^a^ to at least two antimicrobials			
	Pearson’s correlation coefficient	*p*-value (one-tailed)	Number of strains	Number of countries	Pearson’s correlation coefficient	*p*-value (one-tailed)	Number of strains	Number of countries
Estimated total use of antimicrobials (mg/PCU) in 2011^b^	0.507	0.100	956	8	0.672*^c^	0.034	956	8
Use of oral antimicrobials (mg/CPU) in 2011	0.501	0.103	956	8	0.653*	0.040	956	8
Use of injectable antimicrobials (mg/CPU) in 2011	−0.142	0.368	956	8	0.015	0.486	956	8
Proportion of oral antimicrobials (%) in 2011	0.619	0.051	956	8	0.450	0.132	956	8
Proportion of injectable antimicrobials (%) in 2011	−0.644*	0.042	956	8	−0.503	0.102	956	8
Estimated total use of antimicrobials (mg/kg) in 2012^d^	0.540	0.084	956	8	0.694*	0.028	956	8
Estimated total use of antimicrobials (mg/kg) in the mid-1990s^e^	0.808*	0.026	743	6	0.375	0.232	743	6
Use of therapeutic antimicrobials (mg/kg) in the mid-1990s	0.777*	0.035	743	6	0.417	0.205	743	6
Use of growth promoters (mg/kg) in the mid-1990s	0.593	0.107	743	6	0.104	0.422	743	6
Resistance to multiple antimicrobial agents	0.795*	0.009	956	8	1	-	956	8

a Ampicillin resistance is excluded.

b Data from ESVAC report [European Medicines Agency (EMA), 2013].

c Statistically significant (*p* < 0.05) correlations are marked with * symbols.

d Data from JIACRA I report [European Centre for Disease Prevention and Control, European Food Safety Authority, and European Medicines Agency (ECDC, EFSA, and EMA), 2015].

e Data from European Agency for the Evaluation of Medicinal Products (EMEA) (1999).

Correlations between estimated antimicrobial use and observed antimicrobial resistance are summarized in Table 5. Total sales of antimicrobial agents and oral antimicrobial agents in the countries in 2011, calculated from the ESVAC report (European Medicines Agency (EMA), 2013), positively correlated with antimicrobial multiresistance of the *Y. enterocolitica* strains. By contrast, the proportion of injectable antimicrobial agents sold in the countries correlated negatively with antimicrobial resistance. In addition, resistance levels were statistically significantly higher in countries where more than two thirds of antimicrobial sales were orally administered products than in countries where less than two thirds were oral antimicrobials (*p* = 0.015). Total use of antimicrobial agents in 2012, obtained from the JIACRA I report [European Centre for Disease Prevention and Control, European Food Safety Authority, and European Medicines Agency (ECDC, EFSA, and EMA), 2015], did not statistically significantly correlate with antimicrobial resistance but did correlate with antimicrobial multiresistance.

Prevalence of antimicrobial resistance in the studied strains positively correlated with the total sales of antimicrobials and the sales of therapeutic antimicrobials in the mid-1990s, calculated from the report of European Agency for the Evaluation of Medicinal Products (EMEA) (1999). However, the use of growth promoter antimicrobials in the mid-1990s did not significantly correlate with either antimicrobial resistance or multiresistance (Table 5).

## Discussion

We observed wide variation in the antimicrobial resistance profiles of *Y. enterocolitica* 4/O:3 strains in the nine European countries. Alarming antimicrobial resistance levels were found in Spain and Italy, where most strains were resistant to two or more antimicrobials. Similar concerning levels of antimicrobial resistance of *Y. enterocolitica*, including multiresistant strains, have also previously been reported in Italy (Bonardi et al., 2013, 2014, 2016) and Greece (Gousia et al., 2011). Gkouletsos et al. (2019) studied the antimicrobial resistance of Greek *Y. enterocolitica* O:3 strains isolated from production animals, companion animals, and humans, and found no statistically significant differences in the overall levels of antimicrobial resistance between the three groups. As *Y. enterocolitica* strains can spread between production animals, companion animals, and humans, antimicrobial-resistant strains may also spread between these groups. In Spain, trends of increasing antimicrobial resistance to various antimicrobials, including streptomycin, sulfonamides, trimethoprime-sulfamethoxazole, chloramphenicol, and nalidixic acid, have been observed in *Y. enterocolitica* strains isolated from human patients (Prats et al., 2000; Marimon et al., 2017).

According to the ESVAC report [European Medicines Agency (EMA), 2013], tetracyclines were the most common veterinary antimicrobial agents sold for treating food production animals in both Italy and Spain in 2011. The common tetracycline use is also likely to explain the high tetracycline resistance observed in our present study. Tetracyclines were the most sold antimicrobials in Italy in 2018 and, bypassed only by penicillin preparations, the second most sold antimicrobial agents in Spain [European Medicines Agency (EMA), 2020]. Of our study countries, Spain and Italy showed the highest antimicrobial sales for treating food production animals per population correction unit (mg/CFU). Multiresistant strains were frequent in these countries and the observed antimicrobial resistance levels the highest in the present study.

The English *Y. enterocolitica* strains were mainly resistant to sulfamethoxazole, but resistance to trimethoprim, streptomycin, and tetracycline was also observed. According to the ESVAC reports [European Medicines Agency (EMA), 2013, 2020], sulfonamides, following tetracyclines and penicillins, were the third most common veterinary antimicrobial agents sold in the United Kingdom in 2011 and 2018, which may partly explain the relatively high resistance rates to sulfamethoxazole. Sulfamethoxazole is commonly used with trimethoprim, against which antimicrobial resistance was also observed.

Over half of the Belgian strains were resistant to streptomycin, but otherwise antimicrobial resistance levels were moderate in Belgium and Germany. Interestingly, aminoglycosides are not particularly commonly used in Belgium or Germany, yet streptomycin resistance was frequent in Belgium but not in Germany. Low or moderate resistance levels of *Y. enterocolitica* have been reported in Austria, Germany, and Switzerland (Mayrhofer et al., 2004; Baumgartner et al., 2007; Fredriksson-Ahomaa et al., 2007, 2010b; Bucher et al., 2008; von Altrock et al., 2010; Bonke et al., 2011; Meyer et al., 2011; Schneeberger et al., 2015).

The low antimicrobial resistance levels of *Y. enterocolitica* 4/O:3 strains detected in Latvia, Estonia, Finland, and Russia, are in accordance with earlier studies available from these countries (Kontiainen et al., 1994; Terentjeva and Bērziņs, 2010; Bonke et al., 2011). However, Terentjeva and Bērziņs (2010) found high resistance (100%) to sulfamethoxazole in Latvian *Y. enterocolitica* strains and concluded that extensive sulfonamide use for livestock during the time when Latvia belonged to the Soviet Union could have affected the development of such resistance. In our study, resistance to sulfamethoxazole was not observed in the Latvian strains. However, we used the broth microdilution method while Terentjeva and Bērziņs (2010) utilized the disc diffusion method. Different breakpoints and methods complicate the comparison of antimicrobial susceptibility results gained from various studies, and disagreement between different tests is relatively frequent, especially with sulfamethoxazole (Meyer et al., 2011), which may partly explain this difference in results.

The multiresistant *Y. enterocolitica* 4/O:3 strains were most commonly resistant to streptomycin, sulfamethoxazole, and chloramphenicol. This pattern appears to be common in *Y. enterocolitica* strains in several European countries (Baumgartner et al., 2007; Sihvonen et al., 2011; Bonardi et al., 2014, 2016). Karlsson et al. (2021) reported a chromosomally encoded multi-drug resistance cassette containing resistance genes to these three types of antimicrobials and mercury.

Antimicrobial resistance in our study positively and statistically significantly correlated with the total sales of antimicrobials and the sales of therapeutic antimicrobials in the mid-1990s, but no statistically significant correlation was found with growth promoter sales. However, the EU banned the use of antimicrobials as growth promoters in 2006 [European Union (EU), 2005], and most of the antimicrobial growth promoters, such as vancomycin and avoparcin, used in the EU in the past are mainly active against Gram-positive bacteria (Wegener et al., 1999). Despite veterinary antimicrobials currently being prescription-only medicines in the member countries of the ESVAC reports [European Medicines Agency (EMA), 2013, 2020], preventative medications are still commonly used. For example, prophylactic medications are given to healthy animals with no clinical symptoms but with a high risk of disease, while metaphylactic medications are given to healthy animals living in the same group as symptomatic animals. Callens et al. (2012) studied 50 Belgian herds of fattening pigs and found that antimicrobials, including critical and broad-spectrum ones, had been used preventatively in 98% of the herds, and 93% of the group treatments were prophylactic while only 7% were metaphylactic. Along with the therapeutic use of antimicrobials, prophylaxis, and metaphylaxis may be needed in certain situations, for example, if a serious disease is threatening an entire group of animals. However, the benefits of preventative medications should always be considered in relation to the risk of developing antimicrobial resistance.

In the present study, the antimicrobial resistance levels were higher in countries where more than two thirds of antimicrobials were sold in enteral forms, which reflect the importance of using parenteral medications for individual animals rather than enteral mass medications *via* feed. The overall sales data collected by EMA and summarized in Table 4 show that most veterinary antimicrobials in Belgium, Germany, Italy, Spain, and the United Kingdom were sold in enteral forms, such as premixes, oral powders, and oral solutions, while parenteral medications were preferred in Finland, and both enteral and parenteral forms were commonly used in Estonia and Latvia. This finding is supported by Sjölund et al. (2016), who compared antimicrobial use in Belgium, France, Germany, and Sweden and found that the overall use of antimicrobials was highest in German pig herds and lowest in Swedish herds, and antimicrobials were usually given in enteral forms, except in Sweden, where parenteral forms were preferred. According to European Medicines Agency (EMA) (2013), the sales of orally administered antimicrobials are a reasonable estimate of group treatments, because premixes and the majority of oral powders and oral solutions are applicable for group treatment while the sales of small packages of oral powders and oral solutions sufficient for treatment of only a single or a few animals are very low. Frequent use of oral antimicrobials indicates that mass medications are common in certain countries. By contrast, parenteral forms are preferred in Northern Europe [European Medicines Agency (EMA), 2013], which indicates more prudent use of antimicrobial agents mostly targeting individual animals rather than groups of animals.

We observed major differences in veterinary antimicrobial use between the countries. This variation cannot solely be explained by the different proportions of production animal species in European countries, because the antimicrobial use also depends on several other factors, such as the infectious disease situation, economic incentives, and the culture of prescribing antimicrobials [Grave et al., 2012; European Medicines Agency (EMA), 2013, 2020; Speksnijder et al., 2015]. For example, veterinarians in Finland are not allowed to financially profit from selling antimicrobials or other prescription medications. According to guidelines by the European Commission (2015), one key factor in prudent antimicrobial use is to avoid any financial or material benefits for the suppliers or prescribers of medicines.

Some limitations of our study should be considered when interpreting the results. Sales data are an indirect way to simulate the use of antimicrobial agents, as there are no available data on actual antimicrobial use. Dosages vary between and within the classes of antimicrobial agents and between animal species, the proportions of domestic animal species differ by country, and the population correction unit represents all animals, not only pigs. In addition, the population correction unit is a mathematical unit of measurement only and does not represent any actual animal population possibly treated with antimicrobials. Hence, a detailed comparison is difficult, and ESVAC reports should be interpreted carefully [European Medicines Agency (EMA), 2013, 2020]. Despite the limitations of our study and the multifactorial nature of antimicrobial resistance as a phenomenon, the present study shows that the use of antimicrobial agents is a key factor in the emergence of antimicrobial resistance. Our study also shows differences in general antimicrobial policies between the countries. Similarly, Chantziaras et al. (2013) found that antimicrobial use positively correlated with antimicrobial resistance of *E. coli* isolates.

According to the first JIACRA report by European Centre for Disease Prevention and Control, European Food Safety Authority, and European Medicines Agency (ECDC, EFSA, and EMA) (2015), antimicrobials were used more for production animals than for humans in 2011 and 2012 in Europe. However, according to the newest JIACRA report, the situation was reversed in 2016 [European Centre for Disease Prevention and Control, European Food Safety Authority, and European Medicines Agency (ECDC, EFSA, and EMA), 2021]. Despite increasing efforts to reduce antimicrobial use in the EU, the global trends are concerning. For example, Van Boeckel et al. (2015) estimated that antimicrobial consumption in livestock will increase by 67% from 2010 to 2030, mainly because intensive farming is becoming more common in middle-income countries.

To conclude, the antimicrobial resistance of *Y. enterocolitica* 4/O:3 strains of porcine origin varied widely between European countries. Resistance was most frequent in countries where antimicrobials, especially enteral medications, are used in large quantities. The antimicrobial resistance of numerous pathogens, including *Y. enterocolitica*, is considered one of the most severe global health threats. Despite encouraging news that antimicrobial use has generally decreased in Europe during recent years, much work is required globally. We recommend that antimicrobial resistance control should begin already at the farm level. This can be achieved through the strict control of prescriptions, sales, and use of antimicrobial agents. When antimicrobial agents are needed, treating individual animals should be preferred to mass medications whenever possible. Regular antimicrobial susceptibility monitoring and data on actual antimicrobial use are also needed in the battle against antimicrobial resistance.

## Data Availability Statement

The original contributions presented in the study are included in the article/Supplementary Material, further inquiries can be directed to the corresponding author.

## Author Contributions

HK and RK-T designed the study. PO-M, SJ, JK, and MF-A performed the laboratory work and analyzed the antimicrobial resistance data. JK performed the analysis on antimicrobial use. JK and SJ drafted the manuscript. HK, RK-T, MF-A, and PO-M contributed to manuscript revision. All authors contributed to the article and approved the submitted version.

## Funding

This work was partially supported by the Finnish Ministry of Agriculture and Forestry (4877/501/2005) and by the Walter Ehrström Foundation and performed in the Finnish Centre of Excellence in Microbial Food Safety, Academy of Finland (grant numbers 118602 and 141140).

## Conflict of Interest

The authors declare that the research was conducted in the absence of any commercial or financial relationships that could be construed as a potential conflict of interest.

## Publisher’s Note

All claims expressed in this article are solely those of the authors and do not necessarily represent those of their affiliated organizations, or those of the publisher, the editors and the reviewers. Any product that may be evaluated in this article, or claim that may be made by its manufacturer, is not guaranteed or endorsed by the publisher.
