# Supplementary material for: Prudent Antimicrobial Use Is Essential to Prevent the Emergence of Antimicrobial Resistance in Yersinia enterocolitica 4/O:3 Strains in Pigs
Source: Front Microbiol. 2022 Mar 10;13:841841. doi: 10.3389/fmicb.2022.841841 (PMC8967395; doi:10.3389/fmicb.2022.841841)
Supplement: Supplementary file 1 [file Table_1.pdf]

Supplementary Table 1. Number of *Yersinia enterocolitica* 4/O:3 strains studied by country by sampling year.

Country	Number of strains by sampling year									Total
	1999	2000	2001	2002	2003	2004	2005	2006	2007	
Belgium	0	0	0	0	0	0	0	94	0	94
Estonia	0	0	0	0	0	0	143	0	0	143
Finland	0	0	0	50	0	0	0	77	106	233
Germany	14	34	3	8	0	39	0	0	0	98
Italy	0	0	0	0	0	0	0	105	0	105
Latvia	0	0	0	0	0	70	0	0	0	70
Russia	0	0	0	0	0	0	0	53	7	60
Spain	0	0	0	0	0	96	89	0	0	185
UK [a]	0	0	0	0	18	10	0	0	0	28
Total	14	34	3	58	18	215	232	329	113	1016

[a] All the strains from UK originated from England.
