# Supplementary material for: Prudent Antimicrobial Use Is Essential to Prevent the Emergence of Antimicrobial Resistance in Yersinia enterocolitica 4/O:3 Strains in Pigs
Source: Front Microbiol. 2022 Mar 10;13:841841. doi: 10.3389/fmicb.2022.841841 (PMC8967395; doi:10.3389/fmicb.2022.841841)
Supplement: Supplementary file 2 [file Table_2.pdf]

Supplementary Table 2. Minimum inhibitory concentrations (MIC) of porcine *Yersinia enterocolitica* 4/O:3 strains.

Antimicrobial[a]	Country (number of strains)	Percentage of isolates with MIC (µg/µl)[b]										Prevalence of resistant strains in % [c]
Ampicillin		< 0.25	0.25	0.5	1	2	4	8	16	32	> 32	
	Belgium (94)								9.6	87.2	3.2	90.4 (82.1–95.2)
	Estonia (143)									46.9	53.1	100 (96.7–100)
	Finland (233)								7.3	47.6	45.1	92.7 (88.4–95.6)
	Germany (98)									53.1	46.9	100 (95.3–100)
	Italy (105)								22.9	54.3	22.9	77.1 (67.7–84.5)
	Latvia (70)									100		100 (93.5–100)
	Russia (60)								8.3	53.3	38.3	91.7 (80.9–96.9)
	Spain (185)									46.5	53.5	100 (97.5–100)
	UK (28)								3.6	71.4	25.0	96.4 (79.7–99.8)
Cefotaxime		< 0.064	0.064	0.125	0.25	0.5	1	2	>2			
	Belgium (94)	11.7		85.1	3.2							0 (0–4.9)
	Estonia (143)	5.6		88.8	5.6							0 (0–3.3)
	Finland (233)	35.2		63.5	1.3							0 (0–2.0)
	Germany (98)	5.1		83.7	11.2							0 (0–4.7)
	Italy (105)	39		52.4	8.6							0 (0–4.4)
	Latvia (70)			100								0 (0–6.5)
	Russia (60)	40		58.3	1.7							0 (0–7.5)
	Spain (185)	0.5	0.5	91.4	6.5	1.1						0 (0–2.5)
	UK (28)	3.6	17.9	60.7	17.9							0 (0–15.0)
Ceftiofur		< 0.125	0.125	0.25	0.5	1	2	4	8	16	>16	
	Belgium (94)	4.3		91.5	4.3							0 (0–4.9)
	Estonia (143)			89.5	10.5							0 (0–3.3)
	Finland (233)	4.3		71.7	24.0							0 (0–2.0)
	Germany (98)			66.3	32.7	1.0						0 (0–4.7)
	Italy (105)	4.8		77.1	18.1							0 (0–4.4)
	Latvia (70)			100								0 (0–6.5)
	Russia (60)			93.3	6.7							0 (0–7.5)
	Spain (185)			48.1	50.8	0.5	0.5					0 (0–2.5)
	UK (28)			64.3	35.7							0 (0–15.0)

Supplementary Table 1. Continued

Antimicrobial[a]	Country (number of strains)	Percentage of isolates with MIC (µg/µl)[b]										Prevalence of resistant strains in % [c]
Chloramphenicol		< 1	1	2	4	8	16	32	64	128	> 128	
	Belgium (94)	2.1		53.2	30.9	13.8						0 (0–4.9)
	Estonia (143)			9.1	88.1	2.8						0 (0–3.3)
	Finland (233)	0.9		45.9	51.9	1.3						0 (0–2.0)
	Germany (98)			10.2	83.7	6.1						0 (0–4.7)
	Italy (105)			32.4	63.8	2.9	1.0					0 (0–4.4)
	Latvia (70)				95.7	4.3						0 (0–6.5)
	Russia (60)			43.3	51.7	5.0						0 (0–7.5)
	Spain (185)				10.3	0.5			9.2	80.0		89.2 (83.6–93.1)
	UK (28)			32.1	64.3	3.6						0 (0–15.0)
Ciprofloxacin[d]		< 0.008	0.008	0.016	0.032	.064	0.125	0.25	0.5	1	> 1	
	Belgium (94)			79.8	19.1	1.1						0 (0–4.9)
	Estonia (143)			0.7	4.9	93.7	0.7					0 (0–3.3)
	Finland (233)			25.3	60.1	14.6						0 (0–2.0)
	Germany (98)				20.4	79.6						0 (0–4.7)
	Italy (105)	1.9		55.2	41.9	1.0						0 (0–4.4)
	Latvia (70)				18.6	81.4						0 (0–6.5)
	Russia (60)	1.7		8.3	83.3	6.7						0 (0–7.5)
	Spain (185)				17.8	71.9		1.1	9.2			0 (0–2.5)
	UK (28)				28.6	67.9	3.6					0 (0–15.0)
Florfenicol		< 4	4	8	16	32	> 32					
	Belgium (94)	100										0 (0–4.9)
	Estonia (143)	100										0 (0–3.3)
	Finland (233)	98.7		1.3								0 (0–2.0)
	Germany (98)	100										0 (0–4.7)
	Italy (105)	100										0 (0–4.4)
	Latvia (70)	100										0 (0–6.5)
	Russia (60)	100										0 (0–7.5)
	Spain (185)	100										0 (0–2.5)
	UK (28)	100										0 (0–15.0)

Supplementary Table 1. Continued

Antimicrobial[a]	Country (number of strains)	Percentage of isolates with MIC (µg/µl)[b]										Prevalence of resistant strains in % [c]
Gentamicin		< 0.5	.5	1	2	4	8	16	32	64	> 64	
	Belgium (94)	38.3		61.7								0 (0–4.9)
	Estonia (143)	0.7		88.1	11.1							0 (0–3.3)
	Finland (233)	31.8		66.1	2.1							0 (0–2.0)
	Germany (98)			80.6	19.4							0 (0–4.7)
	Italy (105)	26.7		73.3								0 (0–4.4)
	Latvia (70)			100								0 (0–6.5)
	Russia (60)			100								0 (0–7.5)
	Spain (185)	0.5		95.1	4.3							0 (0–2.5)
	UK (28)			57.1	39.3	3.6						0 (0–15.0)
Kanamycin[e]		< 2	2	4	8	16	> 16					
	Belgium (94)	79.8		20.2								0 (0–4.9)
	Estonia (143)	8.4		89.5	2.1							0 (0–3.3)
	Finland (233)	65.2		33.9	0.9							0 (0–2.0)
	Germany (98)	5.1		91.8	3.1							0 (0–4.7)
	Italy (105)	59.0		41.0								0 (0–4.4)
	Latvia (70)	15.7		84.3								0 (0–6.5)
	Russia (60)	86.7		13.3								0 (0–7.5)
	Spain (185)	15.1		84.9								0 (0–2.5)
	UK (28)	7.1		82.1	10.7							0 (0–15.0)
Nalidixic acid		< 1	1	2	4	8	16	32	64	128	> 128	
	Belgium (94)	18.1		77.7	4.3							0 (0–4.9)
	Estonia (143)	0.7		97.9	1.4							0 (0–3.3)
	Finland (233)	15.5		84.0	0.4							0 (0–2.0)
	Germany (98)	4.1		95.9								0 (0–4.7)
	Italy (105)	6.7		89.5	2.9		1.0					0 (0–4.4)
	Latvia (70)			100								0 (0–6.5)
	Russia (60)	5.0		93.3	1.7							0 (0–7.5)
	Spain (185)			89.7							10.3	10.3 (6.5–15.8)
	UK (28)	21.4		67.9	10.7							0 (0–15.0)

Supplementary Table 1. Continued

Antimicrobial[a]	Country (number of strains)	Percentage of isolates with MIC (μg/μl)[b]										Prevalence of resistant strains in % [c]
Streptomycin		< 2	2	4	8	16	32	64	128	256	> 256	
	Belgium (94)			27.7	17.0			1.1	1.1	8.5	44.7	55.3 (44.7–65.4)
	Estonia (143)			3.5	86.7	9.8						0 (0–3.3)
	Finland (233)	0.4		64.8	30.9	3.9						0 (0–2.0)
	Germany (98)	1.0		8.2	72.4	12.2				1.0	5.1	6.1 (2.5–13.3)
	Italy (105)	1.0		22.9	14.3		2.9	1.0	36.2	21.9		61.9 (51.9–71.1)
	Latvia (70)				98.6	1.4						0 (0–6.5)
	Russia (60)			85.0	10.0				1.7	3.3		5.0 (1.3–14.8)
	Spain (185)				2.2				0.5	5.9	91.4	97.8 (94.1–99.3)
	UK (28)			32.1	60.7		7.1					7.1 (1.2–24.9)
Sulfamethoxazole		< 16	16	32	64	128	256	512	1024	2048	>2048	
	Belgium (94)	57.4		13.8	20.2	5.3	1.1				2.1	2.1 (0.4–8.2)
	Estonia (143)	97.2		2.1			0.7					0 (0–3.3)
	Finland (233)	70.0		27.5	2.1				0.4			0.4 (0–2.7)
	Germany (98)	66.3		23.5	6.1						4.1	4.1 (1.3–10.7)
	Italy (105)	9.5		16.2	10.5	2.9				1.0	60.0	61.0 (51.0–70.2)
	Latvia (70)	47.1		21.4	15.7	15.7						0 (0–6.5)
	Russia (60)	65.0		28.3	3.3	1.7		1.7				1.7 (0.1–10.2)
	Spain (185)	1.1									98.9	98.9 (95.7–99.8)
	UK (28)	10.7			7.1						82.1	82.1 (62.4–93.2)
Tetracycline		< 0.5	0.5	1	2	4	8	16	32	64	> 64	
	Belgium (94)			34.0	60.6	1.1			1.1	3.2		4.3 (1.4–11.2)
	Estonia (143)			58.0	42.0							0 (0–3.3)
	Finland (233)			52.8	47.2							0 (0–2.0)
	Germany (98)			41.8	58.2							0 (0–4.7)
	Italy (105)	1.0		10.5	28.6		11.4	7.6	6.7	34.3		48.6 (38.8–58.5)
	Latvia (70)			45.7	54.3							0 (0–6.5)
	Russia (60)			58.3	40.0						1.7	1.7 (0.1–10.2)
	Spain (185)			17.3	37.3	17.8	0.5		3.8	22.7	0.5	27 (20.9–34.1)
	UK (28)			60.7	32.1					7.1		7.1 (1.2–24.9)

Supplementary Table 1. Continued

Antimicrobial[a]	Country (number of strains)	Percentage of isolates with MIC (µg/µl)[b]										Prevalence of resistant strains in % [c]
Trimethophrim		< 0.25	0.25	0.5	1	2	4	8	16	32	> 32	
	Belgium (94)			5.3	44.6	43.6	5.3	1.1				0 (0–4.9)
	Estonia (143)	0.7			0.7	55.2	42.7		0.7			0.7 (0–4.4)
	Finland (233)			3.4	33.5	53.6	9.4					0 (0–2.0)
	Germany (98)					22.4	70.4	7.1				0 (0–4.7)
	Italy (105)				15.2	45.7	8.6				30.5	30.5 (22.1–40.4)
	Latvia (70)				1.4	74.3	24.3					0 (0–6.5)
	Russia (60)			1.7	18.3	73.3	6.7					0 (0–7.5)
	Spain (185)					32.4	64.9	2.7				0 (0–2.5)
	UK (28)				7.1	17.9	42.9	10.7	10.7		10.7	21.4 (9.0–41.4)

[a] Breakpoints used for each antimicrobial agent are marked with vertical lines separating susceptible strains on the left and resistant strains on the right side, respectively. Some antimicrobials have two lines, indicating an additional category of intermediate resistance between the lines.

[b] The studied concentration range for each antimicrobial is marked separately.

[c] Prevalence of resistant strains followed by confidence intervals (95% confidence level).

[d] The breakpoints for ciprofloxacin were outside the tested concentration range (susceptible < 2, intermediate = 2, resistant > 2).

[e] The breakpoints for kanamycin were outside the tested concentration range (susceptible < 32, intermediate = 32, resistant > 32).
